# Site-specific labeling of RNA by combining genetic alphabet expansion transcription and copper-free click chemistry

**DOI:** 10.1093/nar/gkv638

**Published:** 2015-06-29

**Authors:** Tatsuhiko Someya, Ami Ando, Michiko Kimoto, Ichiro Hirao

**Affiliations:** 1 RIKEN Center for Life Science Technologies, 1-7-22 Suehiro-cho, Tsurumi-ku, Yokohama, Kanagawa 230-0045, Japan; 2 TagCyx Biotechnologies, 1-6-126 Suehiro-cho, Tsurumi-ku, Yokohama, Kanagawa 230-0045, Japan; 3 PRESTO, JST, Honcho, Kawaguchi-shi, Saitama 332-0012, Japan

## Abstract

Site-specific labeling of long-chain RNAs with desired molecular probes is an imperative technique to facilitate studies of functional RNA molecules. By genetic alphabet expansion using an artificial third base pair, called an unnatural base pair, we present a post-transcriptional modification method for RNA transcripts containing an incorporated azide-linked unnatural base at specific positions, using a copper-free click reaction. The unnatural base pair between 7-(2-thienyl)imidazo[4,5-*b*]pyridine (Ds) and pyrrole-2-carbaldehyde (Pa) functions in transcription. Thus, we chemically synthesized a triphosphate substrate of 4-(4-azidopentyl)-pyrrole-2-carbaldehyde (N_3_-PaTP), which can be site-specifically introduced into RNA, opposite Ds in templates by T7 transcription. The N_3_-Pa incorporated in the transcripts was modified with dibenzocyclooctyne (DIBO) derivatives. We demonstrated the transcription of 17-, 76- and 260-mer RNA molecules and their site-specific labeling with Alexa 488, Alexa 594 and biotin. This method will be useful for preparing RNA molecules labeled with any functional groups of interest, toward *in vivo* experiments.

## INTRODUCTION

RNA molecules have enormous versatility within living organisms. Structural and biological studies of functional RNA molecules will be facilitated by the site-specific labeling and probing of target RNAs without the loss of activity. The present methods for the chemical synthesis of labeled RNA molecules or post-transcriptional modifications of RNA are very restrictive. Currently, chemical synthesis is limited by the length of the RNA molecule, and post-transcriptional modifications are applicable only to the site-specific 5′- or 3′-terminal labeling of transcripts. In addition, various types of modifications involve immense amounts of time and effort to synthesize each modified component.

Meanwhile, progress in click chemistry has been increasing the feasibility of RNA modifications (1–8). In particular, copper-free click chemistry between an azide compound and a cyclooctyne reagent is becoming very popular (9–12). However, phosphoramidite derivatives containing azide groups are chemically unstable in nucleic acid chemical synthesis (13–15). Thus, a method to embed an azide component into RNA molecules at desired positions could facilitate various modifications with any probes, and thus contributing to functional RNA studies.

**Figure 1.**
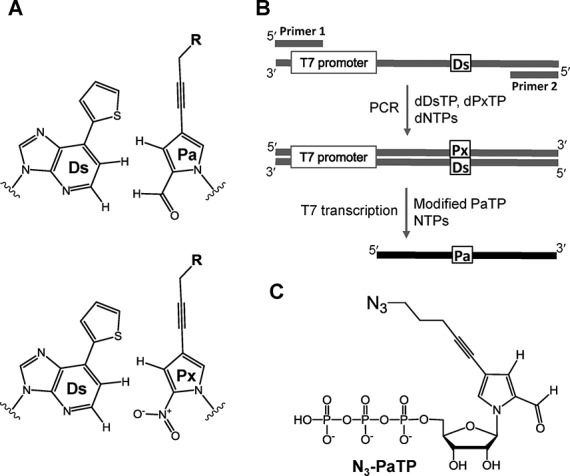
(**A**) Chemical structure of the Ds–Pa pair for T7 transcription and the Ds–Px pair for PCR amplification. (**B**) Scheme of the genetic alphabet expansion for PCR involving the Ds–Px pair and T7 transcription using the Ds–Pa pair. (**C**) Chemical structure of N_3_-PaTP (Compound 6) for transcription.

Recently, genetic alphabet expansion technology using unnatural base pairs has rapidly advanced. By creating an unnatural base pair that functions as a third base pair in replication and transcription, an artificial fifth or sixth base could be introduced into DNA and RNA molecules at desired positions. Over the past 15 years, Benner's, Romesberg's and our group reported several types of unnatural base pairs that function as a third base pair in replication, transcription and/or translation (16–27). Among them, we developed two types of unnatural base pairs, between 7-(2-thienyl)imidazo[4,5-*b*]pyridine (Ds) and 2-nitro-4-propynylpyrrole (Px) (18,19) and between Ds and pyrrole-2-carbaldehyde (Pa), that function in replication and transcription (Figure 1A) (17,28,29). The Ds–Px pair exhibits extremely high efficiency and specificity as a third base pair in replication. However, the nucleotide of Px is relatively unstable under basic conditions, and thus the Ds–Pa pair is useful for the site-specific incorporation of the Pa nucleotide into RNA by transcription, opposite Ds in DNA templates. Thus, the combination of the Ds–Px pair for the preparation of Ds-containing templates by polymerase chain reaction (PCR) with the Ds–Pa pair for transcription enables the site-specific labeling of large RNA molecules (Figure 1B) (29,30). Furthermore, we previously reported that, by attaching an ethynyl group to the Pa base, the triphosphate substrate of the ethynyl-Pa base can be site-specifically incorporated into RNA opposite Ds in DNA templates, by transcription using T7 RNA polymerase (31). The ethynyl-groups in the transcripts can be modified by copper(I)-catalyzed azide-alkyne cycloaddition, using azide derivatives with functional groups. This genetic alphabet expansion method using the ethynyl-Pa base and Ds-containing DNA templates has high potential. However, the click reaction using copper is disadvantageous for subsequent applications of the modified RNA molecules, because the toxic copper contamination of the oligonucleotides impedes *in vivo* applications (32–35).

Here, we present the site-specific labeling of transcripts by the combination of genetic alphabet expansion and copper-free click chemistry. We chemically synthesized a triphosphate of 4-(4-azidopentyl)-pyrrole-2-carbaldehyde (N_3_-Pa) (Figure 1C) and performed the site-specific incorporation of N_3_-PaTP into RNA, opposite Ds in templates, by T7 RNA polymerase. The N_3_-Pa-containing transcripts were then efficiently modified with cyclooctyne-based probes. This method enables the site-specific labeling of large RNA molecules with any probes of interest.

## MATERIALS AND METHODS

### Chemical synthesis

^1^H-, ^13^C- and ^31^P-NMR spectra of compounds dissolved in CDCl_3_, DMSO-*d_6_* or D_2_O were recorded on a BRUKER (300-AVM) magnetic resonance spectrometer. Coupling constant (*J*) values are given in Hz and are correct to within 0.5 Hz. All reagents were purchased from Aldrich, Nacalai Tesque, TCI (Tokyo Chemical Industry Co., Ltd.) and Wako (Wako Pure Chemical Industries, Ltd.). Thin-layer chromatography was performed using TLC Silica Gel 60 F_254_ plates (Merck). Nucleoside derivatives were purified with a Gilson HPLC system using a preparative C18 column (μ-BONDASPHERE, Waters, 19 mm × 150 mm). The triphosphate derivatives were purified by chromatography on a DEAE Sephadex A-25 column (300 mm × 15 mm) and a C18 column (CAPCELL PAK MG III, SHISEIDO, 4.6 mm × 250 mm). High resolution mass spectra (HRMS) and electrospray ionization mass spectra (ESI-MS) were recorded on a Varian 901-MS spectrometer and a Waters micromass ZMD 4000 mass detector equipped with a Waters 2690 LC system, respectively.

### 1-(β-D-Ribofuranosyl)-4-iodopyrrole-2-carbaldehyde (1)

1-(β-D-Ribofuranosyl)-4-iodopyrrole-2-carbaldehyde **1** was synthesized by coupling 4-iodopyrrole-2-carbaldehyde (36) with 2,3,5-tri-*O*-benzyl-D-ribofuranosyl chloride, which was prepared by the chlorination (37) of 2,3,5-tri-*O*-benzyl-D-ribofuranose, using CCl_4_ and tris(dimethylamino)phosphane, followed by a treatment with BBr_3_ (31,17). The structure of **1** was confirmed by nuclear magnetic resonance (NMR) and high resolution mass spectroscopy.

### 1-(2′,3′,5′-*O*-Tri-*tert*-butyldimethylsilyl-β-D-ribofuranosyl)-4-iodopyrrole-2-carbaldehyde (2)

*tert*-Butyldimethylsilyl chloride (TBDMSCl) (1.54 g, 10.20 mmol) was added to a solution of compound **1** (301 mg, 0.85 mmol) and imidazole (1.39 g, 20.40 mmol) in dry pyridine (12 ml). The reaction mixture was stirred at room temperature for 42 h, and the solvent was evaporated. The residue was dissolved in CH_2_Cl_2_, and a saturated NaHCO_3_ solution was added. The organic layer was extracted, dried with Na_2_SO_4_ and evaporated *in vacuo*. The product was purified by silica gel column flash chromatography and eluted by a gradient of CH_2_Cl_2_ (0–100%) in hexane to afford the 1-(2′,3′,5′-*O*-tri-*tert*-butyldimethylsilyl- β-D-ribofuranosyl)-4-iodopyrrole-2-carbaldehyde **2** (529.5 mg, 89%). The structure of **2** was confirmed by ^1^H NMR. ^1^H NMR (CDCl_3_, 300 MHz) δ 9.53 (s, 1H), 7.72 (s, 1H), 7.02 (d, *J* = 1.8 Hz, 1H), 6.52 (d, *J* = 4.8 Hz, 1H), 4.16–4.07 (m, 3H), 3.96 (d, *J* = 11.4 Hz, 1H), 3.78 (d, *J* = 11.7 Hz, 1H), 0.99 (s, 9H), 0.92 (s, 9H), 0.84 (s, 9H), 0.18 (d, *J* = 1.8 Hz, 6H), 0.08 (d, *J* = 1.8 Hz, 6H), −0.02 (s, 3H), −0.16 (s, 3H).

### 1-(2′,3′,5′-*O*-Tri-*tert*-butyldimethylsilyl-β-D-ribofuranosyl)-4-(4-hydroxypentyl)-pyrrole-2-carbaldehyde (3)

Compound **2** (529 mg, 0.76 mmol) was co-evaporated with DMF three times. To a solution of **2**, tetrakis(triphenylphosphine)palladium (Pd(PPh_3_)_4_) (46 mg, 0.04 mmol), CuI (23 mg, 0.12 mmol), trimethylamine (TEA) (159 μl, 1.14 mmol) in DMF (6.2 ml) and 4-pentyn-1-ol (106 μl, 1.14 mmol) were added. The reaction solution was stirred at room temperature for 16 h. The product was evaporated *in vacuo*, dissolved in EtOAc:hexane = 2:1 and washed with brine. After drying with Na_2_SO_4_, the organic extract was evaporated. The residue was purified by silica gel column flash chromatography (eluted by a gradient of MeOH (0–5%) in CH_2_Cl_2_) to give the crude compound **3** (422 mg, 86%). ^1^H NMR (CDCl_3_, 300 MHz) δ 9.51 (s, 1H), 7.85 (s, 1H), 6.94 (d, *J* = 1.8 Hz, 1H), 6.44 (d, *J* = 3.9 Hz, 1H), 4.19–3.97 (m, 4H), 3.81–3.77 (m, 3H), 2.49 (t, *J* = 6.9 Hz, 2H), 1.85 (quin, *J* = 6.6 Hz, 2H), 0.98 (s, 9H), 0.91 (s, 9H), 0.86 (s, 9H), 0.17 (d, *J* = 3.6 Hz, 6H) 0.07 (s, 6H), 0.01 (s, 3H), −0.06 (s, 3H).

### 1-(2′,3′,5′-*O*-Tri-*tert*-butyldimethylsilyl-β-D-ribofuranosyl)-4-(4-azidopentyl)-pyrrole-2-carbaldehyde (4)

To an ice-cold solution of crude compound **3** (404 mg, max 0.62 mmol) in dry CH_2_Cl_2_ (4.1 ml) was added diisopropylethylamine (DIEA) (162 μl, 0.93 mmol). Methanesulfonyl chloride (MsCl) (72 μl 0.93 mmol) was then added to the above solution over a period of 5 min at 0°C. The reaction solution was stirred for 5 h at room temperature. Brine was added to the reaction mixture, which was then extracted with CH_2_Cl_2_. The organic extract was dried with Na_2_SO_4_ and evaporated. The crude mesylated product and NaN_3_ (202 mg, 3.10 mmol) were dissolved in dry DMF (4.1 ml). The reaction mixture was stirred at room temperature for 17 h. The solvent was evaporated, and the product was dissolved in EtOAc:hexane = 2:1, and washed with H_2_O and brine. After drying with Na_2_SO_4_, the organic extract was evaporated. The residue was purified by silica gel column flash chromatography (eluted by a gradient of CH_2_Cl_2_ (0–100%) in hexane) to afford the product **4** (357 mg, 73%, 2 steps total yield). ^1^H NMR (CDCl_3_, 300 MHz) δ 9.51 (s, 1H), 7.86 (s, 1H), 6.95 (s, 1H), 6.44 (d, *J* = 3.9 Hz, 1H), 4.21–4.00 (m, 4H), 3.80 (d, *J* = 11.4 Hz, 1H), 3.45 (t, *J* = 6.6 Hz, 2H), 2.47 (t, *J* = 6.9 Hz, 2H), 1.84 (quin, *J* = 6.9 Hz, 2H), 0.98 (s, 9H), 0.91 (s, 9H), 0.86 (s, 9H), 0.17 (d, *J* = 3.6 Hz, 6H), 0.09 (s, 6H), 0.02 (s, 3H), −0.06 (s, 3H).

### β-D-Ribofuranosyl-4-(4-azidopentyl)-pyrrole-2-carbaldehyde (5)

To a solution of compound **4** (271 mg, 0.40 mmol), a 1 M solution of TBAF in THF (7.5 ml) was added. After 2 h at room temperature, the reaction mixture was evaporated *in vacuo*. The product was purified by silica gel column flash chromatography (eluted by a gradient of MeOH (3–10%) in CH_2_Cl_2_) and RP-HPLC (eluted by a gradient of CH_3_CN (30–80%) in H_2_O) to give compound **5** (102 mg, 78%). ^1^H NMR (DMSO-*d_6_*, 300 MHz) δ 9.51 (s, 1H), 7.91 (s, 1H), 7.11 (s, 1H), 6.33 (d, *J* = 3.9 Hz, 1H), 5.32 (d, *J* = 5.7 Hz, 1H), 5.06 (br s, 2H), 4.01 (br s, 2H), 3.88–3.84 (m, 1H), 3.69–3.51 (m, 2H), 3.45 (t, *J* = 6.9 Hz, 2H), 2.45 (t, *J* = 6.9 Hz, 2H), 1.75 (quin, *J* = 6.9 Hz, 2H). ^13^C NMR (DMSO-*d_6_*, 75 MHz) δ 179.67, 131.24, 130.59, 126.34, 105.90, 89.58, 88.09, 84.56, 75.82, 74.65, 69.36, 60.60, 49.69, 27.52, 16.07. UV-vis spectrum (in EtOH, pH 7.0), *λ*_max_ = 258 nm (*ϵ* = 10.2 × 10^3^), 311 nm (*ϵ* = 8.2 × 10^3^). HRMS (ESI) for C_15_H_18_N_4_NaO_5_ [M+Na]^+^: calcd, 357.1169; found, 357.1170.

### Synthesis of nucleoside 5′-triphosphate (N_3_-PaTP) (6)

To a solution of compound **5** (0.1 mmol) and a proton sponge (33 mg, 0.15 mmol) in trimethyl phosphate (PO(OCH_3_)_3_) (500 μl) was added POCl_3_ (12 μl, 0.13 mmol) at 0°C. The reaction mixture was stirred at 0°C for 4 h. Tri-*n*-butylamine (120 μl, 0.5 mmol) was added to the reaction mixture, followed by 0.5 M bis(tributylammonium)pyrophosphate in a DMF solution (1.0 ml, 0.5 mmol). After 50 min, the reaction was quenched by the addition of 0.5 M triethylammonium bicarbonate (TEAB, 500 μl). The resulting crude product was purified by DEAE Sephadex A-25 column chromatography (eluted by a linear gradient of 50 mM to 1 M TEAB), and then by C18-HPLC (eluted by a gradient of CH_3_CN (20–50%) in 100 mM TEAA) to give compound **6** in a 34% yield from compound **5**. ^1^H NMR (D_2_O, 300 MHz) δ 9.44 (s, 1H), 7.85 (s, 1H), 7.25 (s, 1H), 6.55 (d, *J* = 3.9 Hz, 1H), 4.45 (m, 2H), 4.31–4.25 (m, 3H), 3.51 (t, *J* = 6.6 Hz, 2H), 2.53 (t, *J* = 6.9 Hz, 2H), 1.87 (quin, *J* = 6.9 Hz, 2H). ^31^P NMR (D_2_O, 121 MHz) δ −9.87 (d, *J* = 19.4 Hz, 1P), −10.61 (d, *J* = 19.4 Hz, 1P), −22.47 (t, *J* = 19.4 Hz, 1P). ESI-MS for C_15_H_20_N_4_O_14_P_3_ [M–H]^−^: calcd, 573.00; found, 572.97. UV-vis spectrum (in 10 mM sodium phosphate buffer, pH 7.0), *λ*_max_ = 258 nm (*ϵ* = 10.3 × 10^3^), 311 nm (*ϵ* = 7.6 × 10^3^).

### Biological experimental methods

DNA fragments containing Ds were chemically synthesized with an automated DNA synthesizer (model 392, PerkinElmer Applied Biosystems) or an Oligonucleotide Synthesizer nS-8 (Gene Design) using phosphoramidites of the natural and Ds bases (Glen Research). DNA fragments consisting of only the natural bases were synthesized as described above or purchased from Invitrogen or Gene Design. The chemically-synthesized oligonucleotides were purified by gel electrophoresis.

### DNA templates for T7 transcription

For the 17-mer RNA synthesis, the double-stranded DNA templates (10 μM each of a 35-mer template strand and a 21-mer non-template strand) were annealed in a buffer containing 10 mM Tris-HCl (pH 7.6) and 10 mM NaCl, by heating at 95°C and slow cooling to 4°C. For tRNA synthesis, each 103-mer template DNA was prepared by ligation of a phosphorylated 5′-oligo DNA (48-mer) and non-phosphorylated 3′-oligo DNA (55-mer, control, 35Ds, 47Ds or 59Ds) in the presence of a 70-mer non-template DNA fragment (70-mer), with T4 DNA ligase (Takara), at 16°C for 3 h, and then the ligated 103-mer product was purified by gel electrophoresis. The double-stranded DNA templates (the 103-mer template with the 70-mer non-template) were used for tRNA transcription. The sequences of the DNA fragments were as follows: 5′-GCTCTCCCAACTGAGCTAAATCCGCTATAGTGAGTCGTATTATAGCTT-3′ (5′-oligo, 48-mer), 5′-UmGmGTGCGAATTCTGTGGATCGAACACAGGACCTCCAGATCTTCAGTCTGGC-3′ (3′-oligo control, 55-mer), 5′-UmGmGTGCGAATTCTGTGGATCGAACACAGGACCTCCAGATCTDsCAGTCTGGC-3′ (3′-oligo 35Ds, 55-mer), 5′-UmGmGTGCGAATTCTGTGGATCGAACACAGGDsCCTCCAGATCTTCAGTCTGGC-3′ (3′-oligo 47Ds, 55-mer), 5′-UmGmGTGCGAATTCTGTGGDsTCGAACACAGGACCTCCAGATCTTCAGTCTGGC-3′ (3′-oligo 59Ds, 55-mer), 5′-AAGCTATAATACGACTCACTATAGCGGATTTAGCTCAGTTGGGAGAGCGCCAGACTGAAGATCTGGAGGT-3′ (5′-non-template DNA, 70-mer), where Um = 2′-*O*-methyluridine and Gm = 2′-*O*-methylguanosine. For the 260-mer RNA transcription, a 282-bp double-stranded DNA fragment containing the Ds–Px pair (3′- TDsC - 5′) or a control fragment consisting of natural bases only (3′- TAC - 5′) was prepared as described previously (30).

### T7 transcription

Transcription for the 17-mer RNA was performed in a reaction buffer (20 μl), containing 40 mM Tris-HCl (pH 8.0), 24 mM MgCl_2_, 2 mM spermidine, 5 mM DTT and 0.01% Triton X-100, in the presence of 1 mM natural base substrates (NTPs), 0, 1 or 3 mM N_3_-PaTP, 2 μM DNA template, and 50 U T7 RNA polymerase, unless otherwise indicated. Transcription for the tRNA was performed in the presence of 2 mM natural NTPs, 0, 1 or 2 mM N_3_-PaTP, and 0.5 μM DNA template, and transcription for the 260-mer RNA was performed in the presence of 2 mM natural NTPs, 0, 0.25, 0.5, 1.0 or 2.0 mM N_3_-PaTP, and 0.1 μM DNA template. Internally ^32^P-labeled transcripts for the 17-mer or tRNA were prepared by transcription reactions including 2 μCi [α-^32^P] ATP or 3 μCi [α-^32^P] CTP (PerkinElmer), respectively. To prepare transcripts labeled with ^32^P at the 5′-end, the gel-purified transcripts were labeled with [γ-^32^P]-ATP (PerkinElmer) and T4 polynucleotide kinase (Takara), after treatment with Antarctic phosphatase (New England Biolabs). After transcription at 37°C for 3 h, the reaction was quenched by adding an equal volume of denaturing solution, containing 10 M urea in 1 × TBE. The mixtures were heated at 75°C for 3 min, and the transcripts were purified by denaturing gel electrophoresis (15% PAGE-7 M urea for 17-mer RNA, 10% PAGE-7 M urea for tRNA and 6% PAGE-7 M urea for 260-mer RNA).

### Nucleotide-composition analysis of T7 transcription

The gel-purified transcripts were digested by 0.075 U/μl RNase T2 (Sigma-Aldrich) at 37°C for 2 h, in 15 mM sodium acetate buffer (pH 4.5). The digestion products were analyzed by 2D-TLC, using an HPTLC plate (10 × 10 cm, Merck) with the following developing solvents: isobutyric acid-ammonia-water (66:1:33 v/v/v) for the first dimension, and isopropyl alcohol-HCl-water (70:15:15 v/v/v) for the second dimension. The TLC plates were analyzed with an FLA-7000 bioimager (GE Healthcare). The quantification of each spot was averaged from three data sets.

### Click reaction

The click reaction was performed in reaction buffer (10 μl), containing 200 mM sodium phosphate (pH 7.0), gel-purified transcript RNAs (5 μM for 17-mer RNA and tRNA, 1 μM for 260-mer RNA), and 5–50 molar equivalents of click reagents (Alexa Fluor 488-DIBO alkyne, Alexa Fluor 594-DIBO alkyne or Biotin-DIBO alkyne; Life Technologies). After an incubation at 37°C for 3, 5 or 19 h, the products were separated by gel electrophoresis and analyzed with an FLA-7000 or LAS-4000 bioimager (GE Healthcare).

### Gel mobility shift assay

Biotinylated 260-mer transcripts were detected by gel mobility shift assays, using streptavidin (Promega). Binding experiments were performed in a reaction buffer (10 μl), containing 10 mM Tris-HCl (pH 7.6), 50 mM NaCl, 10 mM EDTA, 2 pmol ^32^P-labeled gel-purified RNA transcript, and 100 pmol streptavidin. After an incubation at 20°C for 1 h, the products were separated by gel electrophoresis and analyzed with an FLA-7000 bioimager.

## RESULTS

### Chemical synthesis of the nucleoside 5′-triphosphate of azide-Pa (N_3_-PaTP)

**Scheme 1.**
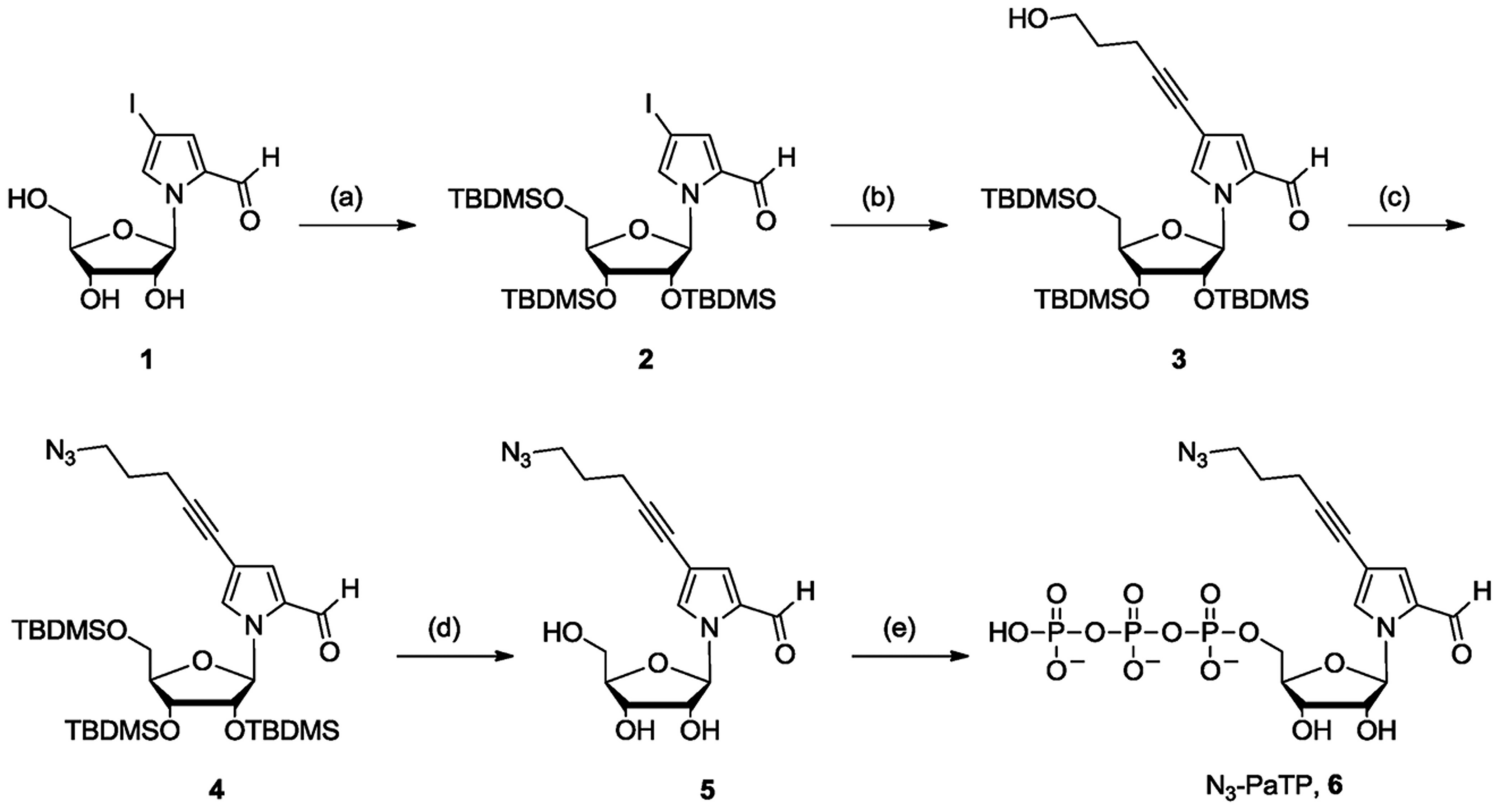
Synthesis of compound **6** (N_3_-PaTP). Reagents: (**a**) TBDMSCl, imidazole in pyridine; (**b**) 4-pentyn-1-ol, CuI, Pd(PPh_3_)_4_, TEA in DMF; (**c**) MsCl, DIEA in CH_2_Cl_2_ then NaN_3_ in DMF; (**d**) TBAF in THF; (**e**) POCl_3_, proton sponge in PO(OCH_3_)_3_ then tri-*n*-butylamine, bis(tributylammonium)pyrophosphate in DMF.

N_3_-PaTP (**6**) was chemically synthesized from 1-(β-D-ribofuranosyl)-4-iodopyrrole-2-carbaldehyde (**1**) in five steps, including the protection of the hydroxyl groups of the sugar moiety, the Sonogashira reaction with 4-pentyn-1-ol, the alcohol conversion to azide and the triphosphorylation (Scheme 1). The total yield of **6** from **1** was ∼14%. The triphosphate **6** was purified by DEAE Sepharose and C18 reversed phase HPLC column chromatographies. The structures and purities of all of the products were confirmed by ^1^H- and/or ^31^P-NMR and ESI-MS (Supplementary Figures S1–S6). The molar absorbance coefficient of N_3_-PaTP (*ϵ* = 10.3 × 10^3^ at 258 nm, *ϵ* = 7.6 × 10^3^ at 311 nm) was determined by the colorimetry of inorganic phosphates using molybdenum blue, after digestion of N_3_-PaTP by calf intestine alkaline phosphatase. N_3_-PaTP was stable under the conditions used for the synthesis, purification and determination of the structural and physical properties.

### T7 transcription using N_3_-PaTP and Ds-containing DNA template (35-mer) and click reaction

**Figure 2.**
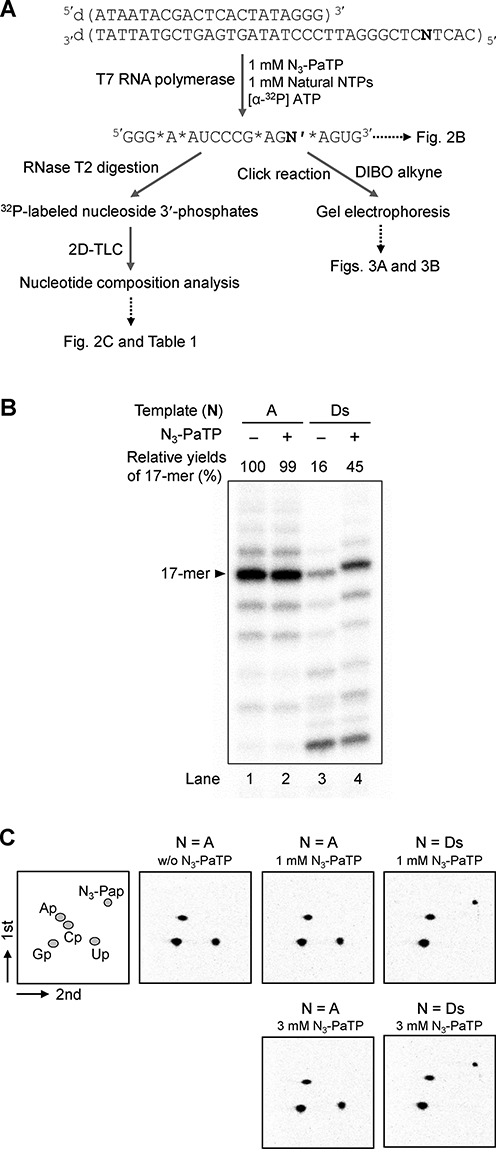
Genetic alphabet expansion transcription for 17-mer RNA. (**A**) Scheme of the experiments. The stars (*) in the sequence shown in Figure 2A indicate the ^32^P-labeled positions. (**B**) Gel electrophoresis of transcripts using 35-mer DNA templates DNA (N = A or Ds) with the natural NTPs (1 mM) and N_3_-PaTP (0 or 1 mM). The transcripts were internally labeled with [α-^32^P] ATP. Relative yields of each transcript were determined by comparison to the yields of native transcripts from a template consisting of the natural bases, and each yield was averaged from three data sets. (**C**) 2D-TLC analysis of labeled ribonucleoside 3′-phosphates obtained by RNase T2 digestion of 17-mer transcripts. Each TLC panel indicates the digestion patterns of transcripts generated from DNA templates (N = A or Ds) in the presence of 0, 1 or 3 mM N_3_-PaTP. The quantification of each experiment is shown in Table 1.

We first examined the selectivity of the incorporation of N_3_-PaTP into RNA opposite Ds in templates by T7 transcription. To determine the accuracy of the selectivity, full-length transcripts (17-mer) were prepared by using 35-mer Ds-containing DNA templates and analyzed by denaturing gel-electrophoresis (17). Transcription was performed by using 1 mM natural base substrates (NTPs) and 0 or 1 mM N_3_-PaTP, including 2 μCi [α-^32^P] ATP, with 2.5 units/μl T7 RNA polymerase at 37°C for 3 h. When using the short Ds-containing DNA templates, the transcription efficiency involving the unnatural base pair is generally lower, by around 45% in this case (Figure 2B, lane 4), relative to that using DNA templates consisting of only the natural bases (Figure 2B, lane 1). As we previously reported (17,29,31), the transcription efficiency with long DNA templates (>50-mer) containing Ds is as high as that with natural base DNA templates, as shown in later. In the absence of N_3_-PaTP, the transcription efficiency was significantly reduced (Figure 2B, lane 3), and thus N_3_-PaTP was predominantly incorporated into RNA, opposite Ds in templates.

For the nucleotide-composition analysis, the transcripts were digested by RNase T_2_ to give nucleoside 3**′**-phosphates (16,17). Since A is incorporated after the unnatural base position in the transcripts, the incorporated N_3_-Pa or misincorporated natural base nucleosides are labeled as 3**′**-^32^P-phosphates by adding [α-^32^P] ATP in transcription (the stars (*) in the sequence shown in Figure 2A indicate the ^32^P-labeled positions). After the digestion of transcripts by RNase T_2_, the labeled nucleoside 3**′**-phosphates were quantitatively analyzed by 2D-TLC (Figure 2C). When using the Ds-containing template, 93% and 96% of N_3_-Pa were incorporated into the transcripts, in the presence of 1 and 3 mM N_3_-PaTP, respectively (Table 1). When using the template consisting of only the natural bases, no N_3_-Pa was observed in the digested products, even in the transcription using 3 mM N_3_-PaTP. Therefore, N_3_-PaTP was selectively incorporated opposite Ds in the template.

**Table 1.** Nucleotide-composition analysis of 17-mer transcripts

Entry	Template (N)^a^	N_3_-PaTP (mM)	Composition of nucleotides incorporated at 5′ neighbor of A				
			Ap	Gp	Cp	Up	N_3_-Pap
1	N = A	0	0.99^b^ [1]^c^ (<0.01)^d^	1.95 [2] (<0.01)	0.01 [0] (<0.01)	1.04 [1] (<0.01)	n.d.^e^ [0] (-)
2		1	1.04 [1] (<0.01)	1.96 [2] (<0.01)	0.01 [0] (<0.01)	1.00 [1] (<0.01)	0.01 [0] (<0.01)
3		3	1.01 [1] (<0.01)	1.93 [2] (<0.01)	0.01 [0] (<0.01)	1.03 [1] (<0.01)	0.02 [0] (<0.01)
4	N = Ds	1	1.02 [1] (<0.01)	2.03 [2] (<0.01)	0.01 [0] (<0.01)	0.02 [0] (<0.01)	0.93 [1] (<0.01)
5		3	1.02 [1] (<0.01)	2.00 [2] (<0.01)	0.01 [0] (<0.01)	0.01 [0] (<0.01)	0.96 [1] (<0.01)

^a^The sequence of each DNA template: 5′-CACT**N**CTCGGGATTCCCTATAGTGAGTCGTATTAT-3′.

^b^The values were determined from the following formula: (radioactivity of each nucleotide)/[total radioactivity of all nucleotides (3′-monophosphates)] × (total number of nucleotides at 5′-neighbor positions of the [α-^32^P] ATP incorporation sites).

^c^The theoretical number of each nucleotide is shown in brackets.

^d^Standard deviations are shown in parentheses.

^e^Not detected.

**Figure 3.**
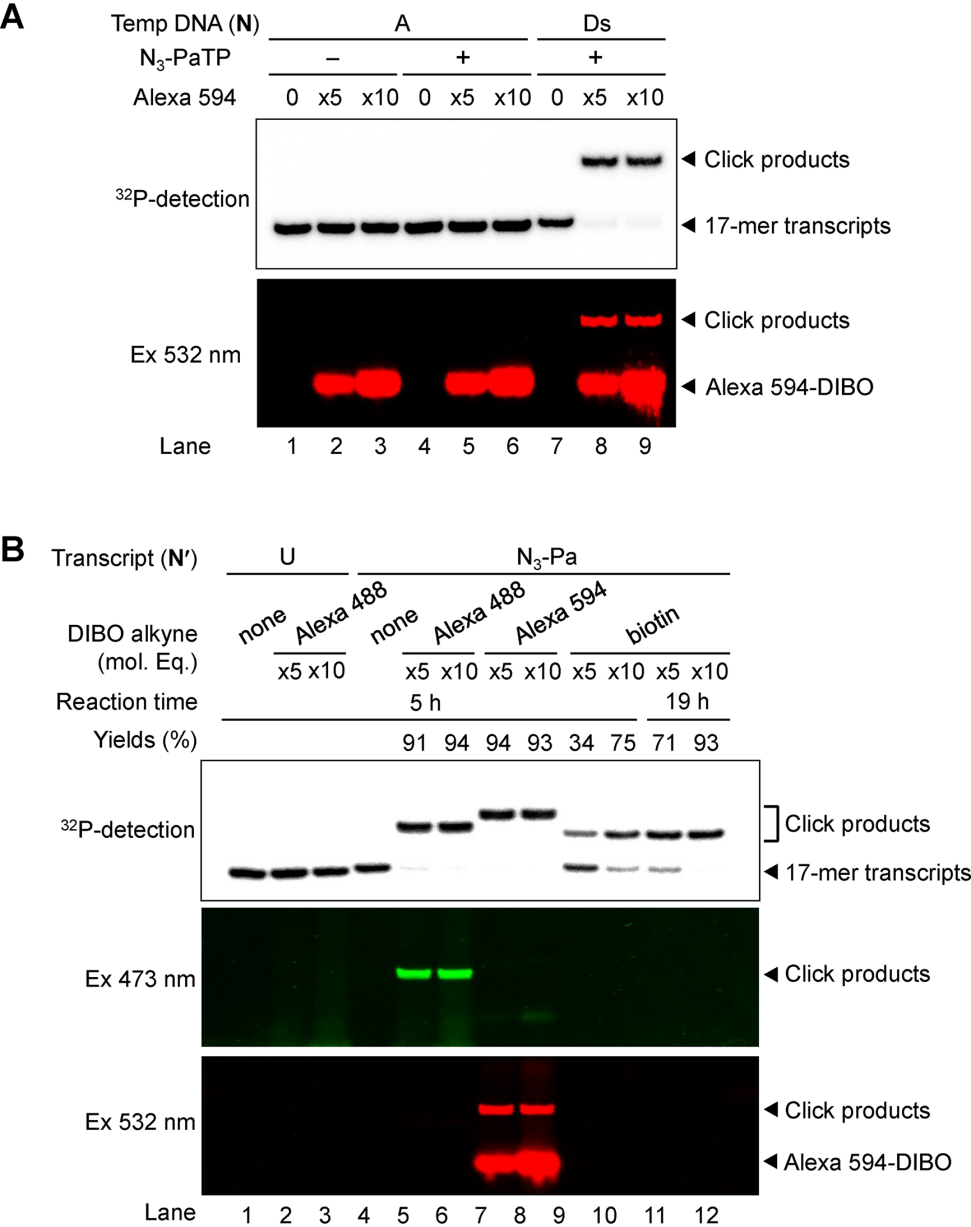
Post-transcriptional labeling of 17-mer transcripts containing N_3_-Pa, by the copper-free click reaction. (**A**) Gel-electrophoresis of Alexa 594-labeled ^32^P-RNA (17-mer) transcribed from DNA templates (N = A or Ds) with the natural NTPs (1 mM) and N_3_-PaTP (0 or 1 mM). Each transcript (5 μM) was modified with Alexa 594-DIBO (25 or 50 μM) at 37°C for 5 h. Products were fractionated on a 20% denaturing polyacrylamide gel containing 7 M urea and were detected by their radioactivity (^32^P-detection) or fluorescence (Ex 532 nm), using an FLA-7000 imager in the Cy3 mode (excitation 532 nm/emission filter O580). (**B**) Post-transcriptional modification of 17-mer transcripts containing N_3_-Pa with Alexa 488, Alexa 594 or biotin reagent. ^32^P-labeled 17-mer transcripts (5 μM) were modified with each click reagent (25 or 50 μM Alexa 488-DIBO, Alexa 594-DIBO or Biotin-DIBO) at 37°C for 5–19 h. Products were fractionated on a 20% denaturing polyacrylamide gel containing 7 M urea and were detected by their radioactivity (^32^P-detection) or fluorescence (Ex 473 nm for Alexa 488 and Ex 532 nm for Alexa 594), using an FLA-7000 imager in the FAM mode (excitation 473 nm/emission filter Y520) and the Cy3 mode. The amounts of click products (yields) were determined from the band intensities, and each yield was averaged from three data sets.

Next, we examined the copper-free click reaction of the transcripts (17-mer), using the fluorescent Alexa Fluor 594-DIBO Alkyne (the dibenzocyclooctyne derivative of Alexa 594, Alexa 594-DIBO) (Figure 2A). For the click reaction, we used the transcripts obtained by T7 transcription in the presence of 1 mM N_3_-PaTP and NTPs. The transcripts (5 μM) were treated with 5 or 10 molar equivalents of Alexa 594-DIBO, in 200 mM sodium phosphate buffer (pH 7.0) at 37°C for 5 h. The products were analyzed by gel-electrophoresis (Figure 3A). Both the reacted and unreacted transcripts were identified from their radioactivities on the gel (^32^P-detection in Figure 3A), and Alexa 488 was detected by 532-nm excitation (Ex 532 nm in Figure 3A). Most of the transcripts were reacted with at least five molar equivalents of the DIBO reagent (lane 8 in Figure 3A), and no products were observed from the transcripts obtained by using the natural-base template in the presence of N_3_-PaTP (lanes 5 and 6 in Figure 3A), confirming that N_3_-PaTP is rarely misincorporated into RNA opposite the natural bases in templates. From the ^32^P-detection band densities of the gel, around 93% of the transcripts were reacted with five molar equivalents of the DIBO reagent. Since the incorporation selectivity of N_3_-PaTP was 93% when using 1 mM N_3_-PaTP and NTPs, all of the N_3_-Pa in the transcripts could be modified.

We also examined the copper-free click reaction of the 17-mer transcripts with Alexa 488-DIBO and biotin-DIBO (Figure 3B). Alexa 488 was detected by 473-nm excitation. The reactivity of biotin-DIBO was lower than those of Alexa 488- and 594-DIBO, and increasing the molar equivalents (10 eq.) of the DIBO reagent and the reaction time (19 h) improved the reactivity (lane 12 in Figure 3B).

### Site-specific labeling of tRNA molecules containing N_3_-Pa by the copper-free click reaction

**Figure 4.**
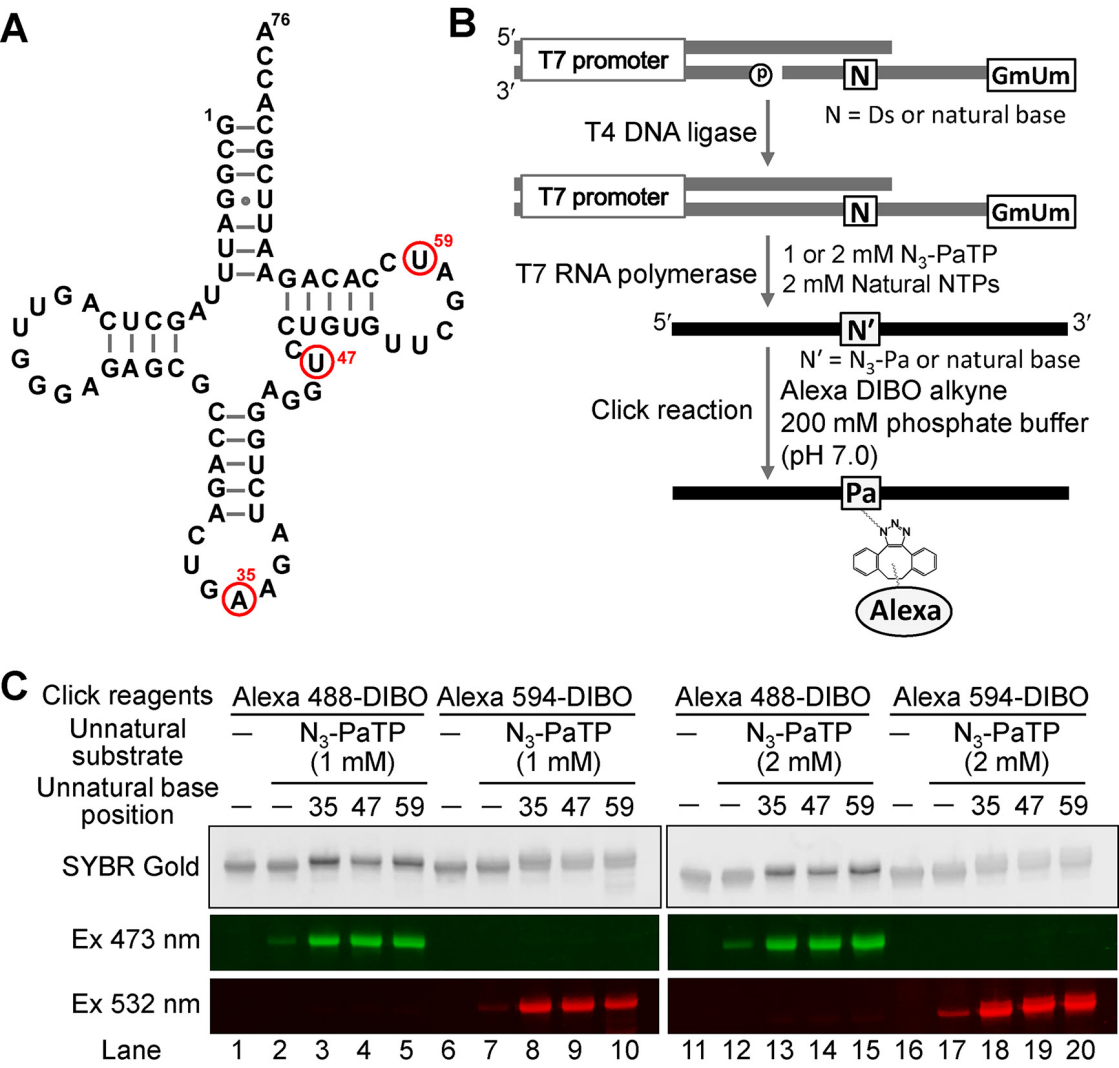
Post-transcriptional labeling of tRNA transcripts (76-mer) containing N_3_-Pa at position 35, 47 or 59, by the copper-free click reaction. (**A**) The secondary structure of yeast tRNA^Phe^. Red circles indicate the incorporation positions of N_3_-Pa. (**B**) Scheme of template preparation, T7 transcription and modification. (**C**) Gel-electrophoresis of each fluorescently labeled transcript generated from templates in the presence of natural NTPs (2 mM) and N_3_-PaTP (0, 1 or 2 mM). Each transcript (5 μM) was labeled with click reagents (50 μM Alexa 488-DIBO or Alexa 594-DIBO) at 37°C for 5 h. Products were analyzed with an FLA-7000 imager in the FAM mode and the Cy3 mode.

Since we confirmed the high incorporation selectivity of N_3_-PaTP and the high reactivity of N_3_-Pa for the copper-free click reaction using short RNA fragments (17-mer), we next performed the site-specific labeling of a tRNA molecule, as a longer transcript (76-mer). To incorporate N_3_-Pa specifically at positions 35, 47 and 59 in yeast tRNA^Phe^ transcripts, we prepared each DNA template containing Ds (35Ds, 47Ds and 59Ds) by ligation, using chemically synthesized DNA fragments. We introduced 2′-*O*-methyl-ribonucleosides at two positions from the 5′-termini of the template strands, to reduce the by-products obtained by non-templated nucleotide additions during T7 transcription (38). Transcription was performed in the presence of 0, 1 or 2 mM N_3_-PaTP and 2 mM NTPs. After purification by denaturing gel-electrophoresis, the transcripts were reacted with 10 molar equivalents of each DIBO reagent, Alexa 488-DIBO or Alexa 594-DIBO, at 37°C for 5 h, and the fluorescently-labeled products were fractionated on a gel and analyzed (Figure 4).

**Figure 5.**
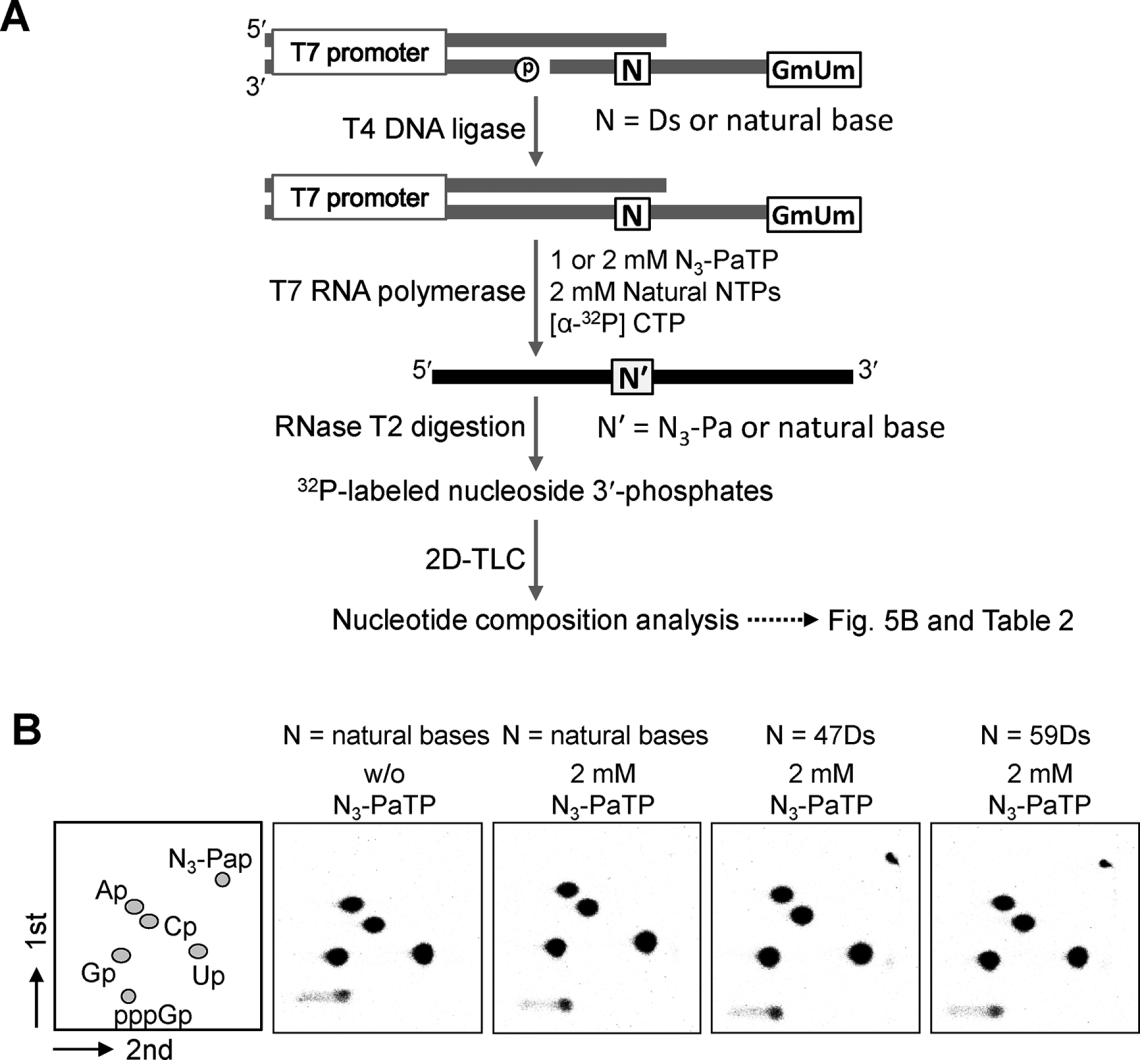
Nucleotide composition analysis by 2D-TLC of ^32^P-labeled ribonucleoside 3′-phosphates, obtained from RNase T2 digestion of tRNA transcripts. (**A**) Scheme of the experiments. (**B**) Transcripts were internally labeled with [α-^32^P] CTP. Each TLC panel indicates the digestion patterns of transcripts generated from DNA templates (N = A or Ds) in the presence of 0 or 2 mM N_3_-PaTP. The quantification of each experiment is shown in Table 2.

Each position of the tRNA was effectively labeled with Alexa 488 and Alexa 594. The labeling efficiencies of each transcript obtained by using 2 mM N_3_-PaTP (lanes 13–15 and 18–20 in Figure 4) were higher than those obtained by using 1 mM N_3_-PaTP (lanes 3–5 and 8–10 in Figure 4). Slight misincorporations of N_3_-PaTP opposite the natural bases were also observed (lanes 2, 7, 12 and 17 in Figure 4). To determine the precise incorporation selectivity of N_3_-PaTP at different concentrations (1 or 2 mM) during transcription, the transcripts obtained using the 47Ds and 59Ds templates were subjected to nucleotide-composition analyses (Figure 5 and Table 2). When using 1 mM N_3_-PaTP, the incorporation selectivity was around 85–86%, and when using 2 mM N_3_-PaTP, the incorporation selectivity increased to around 93–95%. However, the misincorporation rates were also increased with the higher concentration of N_3_-PaTP (Table 2), as shown in lanes 2, 7, 12 and 17 in Figure 4. The misincorporation rates per base were 0.059% and 0.12% for transcription reactions in the presence of 1 mM and 2 mM N_3_-PaTP, respectively.

**Table 2.** Nucleotide-composition analysis of tRNA transcripts

Entry	Template	N_3_-PaTP (mM)	Composition of nucleotides incorporated at 5′ neighbor of C				
			Ap	Gp	Cp	Up	N_3_-Pap
1	control	0	2.98^a^ [3]^b^ (<0.01)^c^	4.01 [4] (<0.01)	3.98 [4] (<0.01)	6.03 [6] (0.02)	n.d.^e^ [0] (-)
2	control	1	2.99 [3] (0.02)	4.05 [4] (<0.01)	3.92 [4] (0.01)	6.02 [6] (0.01)	0.01 [0] (<0.01)
3	47Ds	1	3.04 [3] (<0.01)	4.04 [4] (<0.01)	4.00 [4] (<0.01)	5.07 [5] (<0.01)	0.86 [1] (<0.01)
4	59Ds	1	3.03 [3] (<0.01)	4.08 [4] (<0.01)	3.96 [4] (<0.01)	5.09 [5] (<0.01)	0.85 [1] (<0.01)
5	control	0	2.96 [3] (0.01)	4.04 [4] (0.01)	3.93 [4] (0.01)	6.06 [6] (0.01)	n.d. [0] (-)
6	control	3	2.97 [3] (0.01)	4.05 [4] (0.01)	3.95 [4] (0.01)	6.02 [6] (0.01)	0.02 [0] (0.01)
7	47Ds	3	2.99 [3] (<0.01)	4.08 [4] (0.01)	3.95 [4] (0.03)	5.05 [5] (0.01)	0.93 [1] (0.01)
8	59Ds	3	2.98 [3] (0.02)	4.06 [4] (0.01)	3.93 [4] (0.01)	5.08 [5] (<0.01)	0.95 [1] (0.02)

^a^The values were determined from the following formula: (radioactivity of each nucleotide)/[total radioactivity of all nucleotides (3′-monophosphates)] × (total number of nucleotides at 5′-neighbor positions of the [α-^32^P] CTP incorporation sites).

^b^The theoretical number of each nucleotide is shown in brackets.

^c^Standard deviations are shown in parentheses.

^d^Not detected.

### Transcription and site-specific labeling of the 260-mer RNA

**Figure 6.**
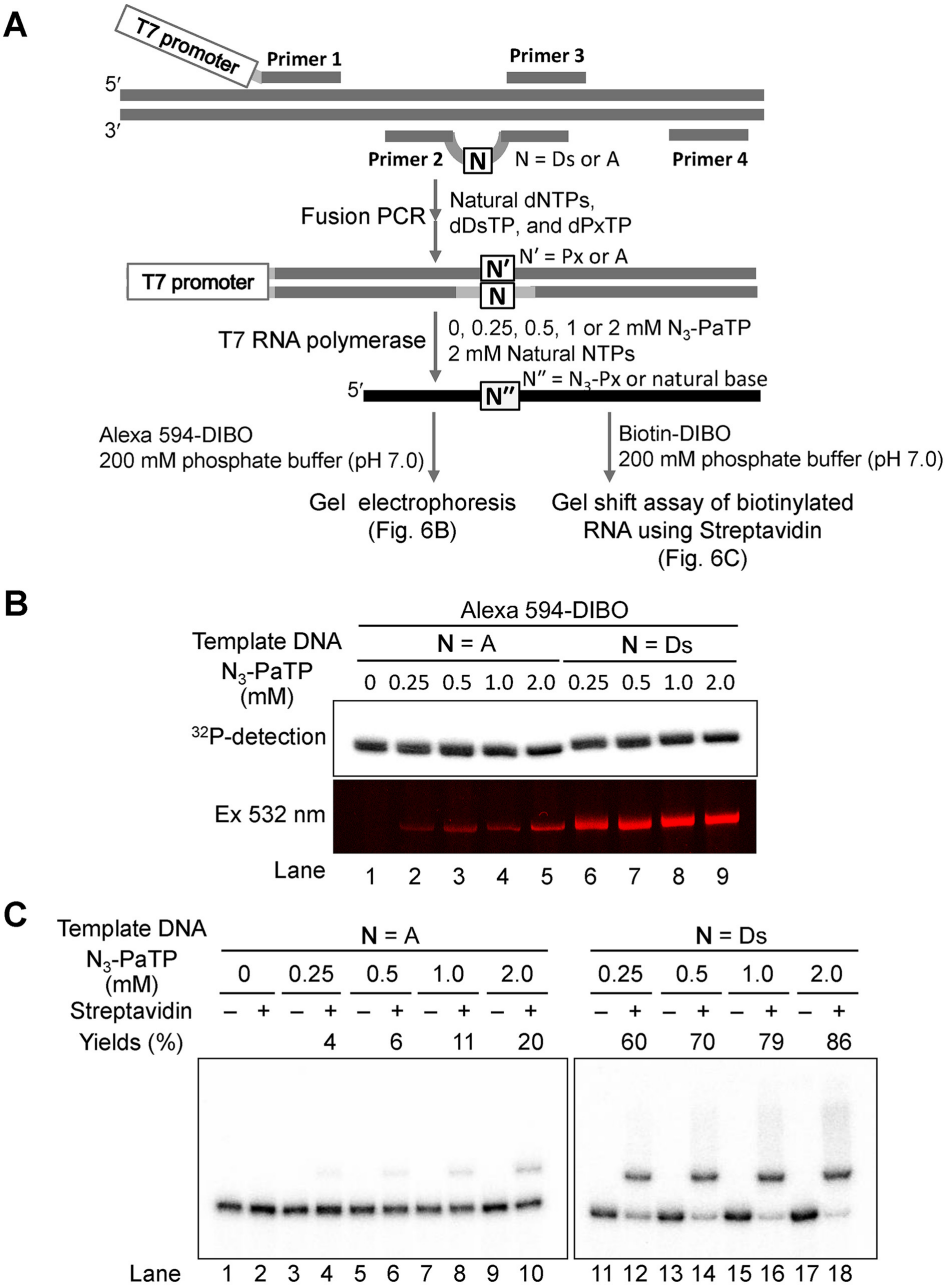
Transcription and site-specific modification of a long RNA (260-mer) containing N_3_-Pa. (**A**) Scheme of DNA template preparation by fusion PCR, T7 transcription and modification by the copper-free click reaction. (**B**) Gel-electrophoresis of ^32^P-labeled 260-mer RNA transcribed from templates in the presence of the natural NTPs (2 mM) and N_3_-PaTP (0, 0.25, 0.5, 1, or 2 mM), after the click reaction with 50 μM Alexa 594-DIBO in 200 mM sodium phosphate (pH 7.0) at 37°C for 5 h. Products were detected with an FLA-7000 imager in the Cy3 mode. (**C**) Gel-shift analysis of ^32^P-labeled 260-mer transcripts (1 μM) modified with Biotin-DIBO (50 μM) at 37°C for 19 h. Products (0.2 μM) were mixed with streptavidin (10 μM), incubated at 20°C for 1 h, and then analyzed on a 6% denaturing polyacrylamide gel. The amounts of the complex (yields) between biotinylated transcripts and streptavidin were determined from the band intensities, and each yield was averaged from three data sets.

To demonstrate the wide range of applications of this labeling method, we examined the transcription of a much longer RNA molecule (260-mer) and its specific labeling at position 43. For the transcription, a 282-bp double-stranded DNA template containing Ds was prepared by fusion PCR, involving another set of an unnatural base pair between Ds and Px that exhibits high fidelity in PCR, using a plasmid DNA (Figure 6A) (30). To evaluate the misincorporation of N_3_-PaTP opposite natural bases in the long template, we also prepared a control 282-bp double-stranded DNA template consisting of only the natural base pairs. T7 transcription was performed using these templates with different concentrations (0, 0.25, 0.5, 1.0 or 2.0 mM) of N_3_-PaTP and 2 mM natural NTPs, at 37°C for 3 h. After transcription, the transcripts were purified by denaturing gel electrophoresis. The 5**′**-phosphate of the transcripts was removed by phosphatase, and then the 5**′**-terminus of each transcript was ^32^P-labeled with T4 polynucleotide kinase and [γ-^32^P] ATP, for further analysis.

The ^32^P-labeled transcripts (1 μM) were reacted with 50 molar equivalents of DIBO reagents (Alexa 594-DIBO or biotin-DIBO) at 37°C for 5 h (for Alexa 594-DIBO) or for 19 h (for biotin-DIBO), and the modified transcripts were analyzed by denaturing gel electrophoresis (Figure 6B for Alexa 594 modification and Figure 6C for biotinylation). The presence of higher concentrations of N_3_-PaTP during the transcription of the Ds-containing DNA template increased the labeling efficiency with Alexa 594-DIBO (lanes 6–9 in Figure 6B). Simultaneously, the misincorporation rates of N_3_-PaTP opposite the natural bases also increased with the higher N_3_-PaTP concentrations, as shown in the Alexa 594-DIBO modification of the transcripts from the control DNA template (lanes 1–5 in Figure 6B).

To estimate the incorporation selectivity of N_3_-PaTP opposite Ds and the misincorporation rate of N_3_-PaTP opposite the natural bases in transcription, we quantified the biotin-labeled transcripts prepared with biotin-DIBO, by gel-shift assays using streptavidin (Figure 6C). When using 0.25, 0.5, 1.0 and 2.0 mM N_3_-PaTP, the selectivities of the N_3_-Pa incorporation opposite Ds were 60%, 70%, 79% and 86%, respectively (lanes 11–18 in Figure 6C), and the misincorporation rates were 0.015%, 0.023%, 0.042% and 0.077% per base, respectively (lanes 1–10 in Figure 6C). At a glance, the selectivity and misincorporation rates were lower than those of the tRNA transcripts. This might be because of the low reactivity of biotin-DIBO. Thus, even for long RNA molecules, the Ds and N_3_-Pa base pair system functions with high selectivity in transcription and post-transcriptional modification by the copper-free click reaction.

## DISCUSSION

We developed a site-specific post-transcriptional modification method by the genetic alphabet expansion system, using the Ds–Pa pair combined with copper-free click chemistry. The chemically synthesized nucleoside 5′-triphosphate of the azide-conjugated unnatural Pa base was very stable under physiological conditions, and was efficiently and selectively incorporated into RNA opposite Ds in templates by T7 transcription. The incorporated N_3_-Pa in transcripts can be modified by copper-free click chemistry with any DIBO derivatives. Due to the difficulties of phosphoramidite synthesis with azide-nucleoside derivatives (13–15), the chemical synthesis of RNA containing azide groups is very limited. Thus, our unnatural base pair system would be very useful for the site-specific incorporation of azide groups into RNA. Furthermore, long RNA molecules (more than 200 bases) could be modified by this system, within the constraint of the base pairing fidelity. For the transcription of long RNA molecules, their DNA templates can be prepared and amplified by PCR involving the Ds–Px pair, as shown in Figure 6A.

When using an equivalent molar ratio of the natural and N_3_-Pa base substrates, the incorporation selectivity of N_3_-PaTP opposite Ds is 85–86% and the misincorporation rate of N_3_-PaTP opposite the natural bases is 0.059% per base. This fidelity is very similar to our previous data for the biotinylated PaTP, with 90% selectivity and a 0.059% misincorporation rate (17). Furthermore, these unnatural base misincorporation rates are lower than those of the non-cognate pairings among the natural bases. Our previous data for the misincorporation rate of biotinylated uridine 5'-triphosphate (UTP) opposite the natural bases, except for A, showed 0.138% per base in T7 transcription under similar conditions (17). This natural base misincorporation rate is equivalent to that (0.12% per base) in the transcription using 2 mM N_3_-PaTP and 2 mM natural NTPs, in which the selectivity of N_3_-PaTP opposite Ds increases to 93–95%. Thus, depending on the desired objectives, the molar ratio between the unnatural and natural base substrates should be adjusted. If the modification efficiency is important, then the combination of 2 mM N_3_-PaTP and 2 mM natural base NTPs should be chosen. In contrast, if a single modification is required, such as for analyzing a single RNA molecule, then reducing the N_3_-PaTP concentration is recommended.

The ability to incorporate a new variable unit into biopolymers will stimulate researchers’ imagination. Fluorescently-labeled RNA molecules are useful to examine interactions with other fluorescently-labeled molecules, such as tRNA-ribosome and tRNA-rRNA interactions in translation, by fluorescence resonance energy transfer (FRET) in an *in vitro* system (39). The injection of labeled RNA molecules into a cell will allow observations of the localization of functional RNA molecules at the single cell level (40). However, our method is still just shy of being applicable to single-molecule analyses using labeled large RNA molecules, like rRNA, because of the slight misincorporation of N_3_-Pa opposite natural bases.

An advantage of the unnatural base pair system is that it can be applied to *in vivo* experiments. Recently, Romesberg's group demonstrated the introduction of an artificial DNA containing their unnatural base pair into *Escherichia coli* (24). Thus, our RNA modification method could be used for analyzing the localization of target RNA molecules that are expressed in a cell: by transcription with N_3_-PaTP using a cell in which the Ds–Px pair is introduced into a specific position of a target gene, the target transcripts modified with N_3_-Pa could be observed by histological staining using DIBO fluorescence. To realize this *in vivo* imaging, an efficient incorporation method of fluorescent click chemistry reagents into cells without any negative effects should be developed in the future.

We now have two sets of labeled RNA molecules, in which one has N_3_-Pa at a desired position and another has ethynyl-linked Pa (Eth-C4-Pa) that we previously reported (31). The combination of N_3_-Pa and Eth-C4-Pa in two RNA molecules might be fixed by clicking each other through the intermolecular RNA-RNA interaction. By using the mixture of N_3_-PaTP and Eth-C4-PaTP in transcription, the tertiary structure of a single RNA molecule could be fixed between two incorporation sites with a 50% chance if the sites between N_3_-Pa and Eth-C4-Pa are spatially close each other.

Another type of double-labeling in a single RNA molecule is also possible by this Ds–Pa pair transcription system. Modified Ds bases, as well as Ds, can be incorporated into a specific position of transcripts, opposite Pa in templates, by T7 transcription (41,42). One of the modified Ds bases is a strongly fluorescent 7-(2,2’-bithien-5-yl)-imidazo[4,5-*b*]pyridine (Dss) base (excitation max.: 370 nm, emission max.: 442 nm) (41,43), and FRET experiments of functional RNA molecules can be performed by site-specific double-labeling with Dss and other fluorescent groups with an excitation range of 440–550 nm, such as Cy3 and Alexa 555, which can be introduced into an N_3_-Pa position in transcripts. Another unnatural base, 2-amino-6-thienylpurine (s), is also fluorescent (excitation max.: 352 nm, emission max.: 434 nm) and can be incorporated into RNA opposite Pa by T7 transcription (42,44). The double-labeling of functional RNA molecules by the combination of fluorescent-Pa and s is in progress.

## Supplementary Material

SUPPLEMENTARY DATA
