# Supplementary material for: Site-specific labeling of RNA by combining genetic alphabet expansion transcription and copper-free click chemistry
Source: Nucleic Acids Res. 2015 Jun 29;43(14):6665–76. doi: 10.1093/nar/gkv638 (PMC4538826; doi:10.1093/nar/gkv638)
Supplement: SUPPLEMENTARY DATA [file supp_43_14_6665__index.html]

Site-specific labeling of RNA by combining genetic alphabet expansion transcription and copper-free click chemistry — SUPPLEMENTARY DATA 

# Site-specific labeling of RNA by combining genetic alphabet expansion transcription and copper-free click chemistry

## SUPPLEMENTARY DATA

- SUPPLEMENTARY DATA
