# Supplementary material for: Site-specific labeling of RNA by combining genetic alphabet expansion transcription and copper-free click chemistry
Source: Nucleic Acids Res. 2015 Jun 29;43(14):6665–76. doi: 10.1093/nar/gkv638 (PMC4538826; doi:10.1093/nar/gkv638)
Supplement: SUPPLEMENTARY DATA [file supp_gkv638_nar-00991-y-2015-File003.pdf]

Site-specific labeling of RNA by combining genetic alphabet expansion transcription and copper-free click chemistry

Tatsuhiko Someya[1], Ami Ando[1], Michiko Kimoto[1, 2, 3], Ichiro Hirao[1,2,*]

[1] RIKEN Center for Life Science Technologies, 1-7-22 Suehiro-cho, Tsurumi-ku, Yokohama, Kanagawa 230-0045, Japan

[2] TagCyx Biotechnologies, 1-6-126 Suehiro-cho, Tsurumi-ku, Yokohama, Kanagawa 230-0045, Japan

[3] PRESTO, JST, Honcho, Kawaguchi-shi, Saitama 332-0012, Japan

* To whom correspondence should be addressed. Tel: +81-45-503-9644; Fax: +81-45-503-9645; Email: ihirao@riken.jp

Supplementary data

NMR and MS spectra of compounds.

Figure S1 ^{1}H NMR spectrum of compound **2**.

Figure S2 ^{1}H NMR spectrum of compound **3**.

Figure S3 ^{1}H NMR spectrum of compound **4**.

Figure S4 ^{1}H and ^{13}C NMR spectra of compound **5**.

Figure S5 ^{1}H and ^{31}P NMR spectra of compound **6**.

Figure S6 DEAE column elution pattern and HRMS spectrum of compound **6**.

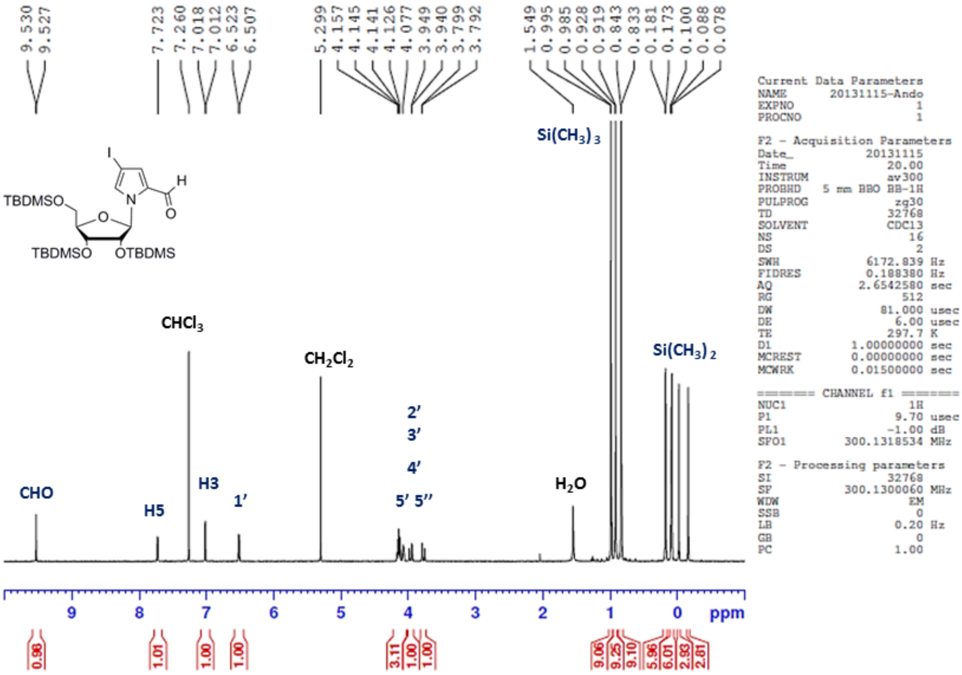

Figure S1. ^{1}H NMR spectrum of compound **2**

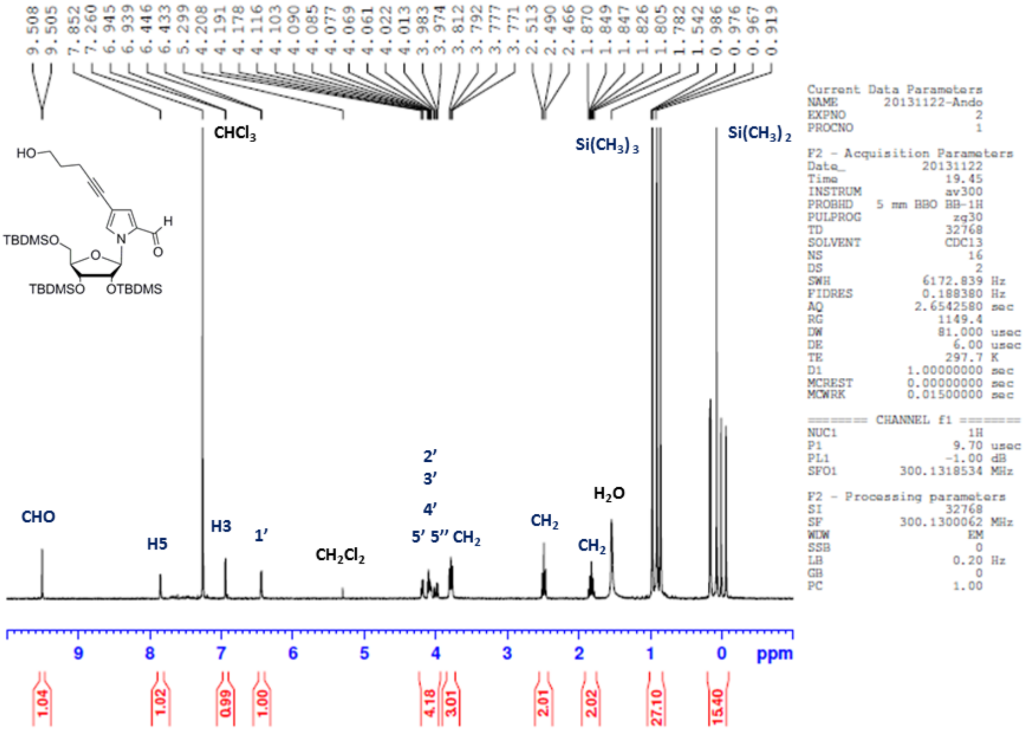

Figure S2. ^{1}H NMR spectrum of compound **3**.

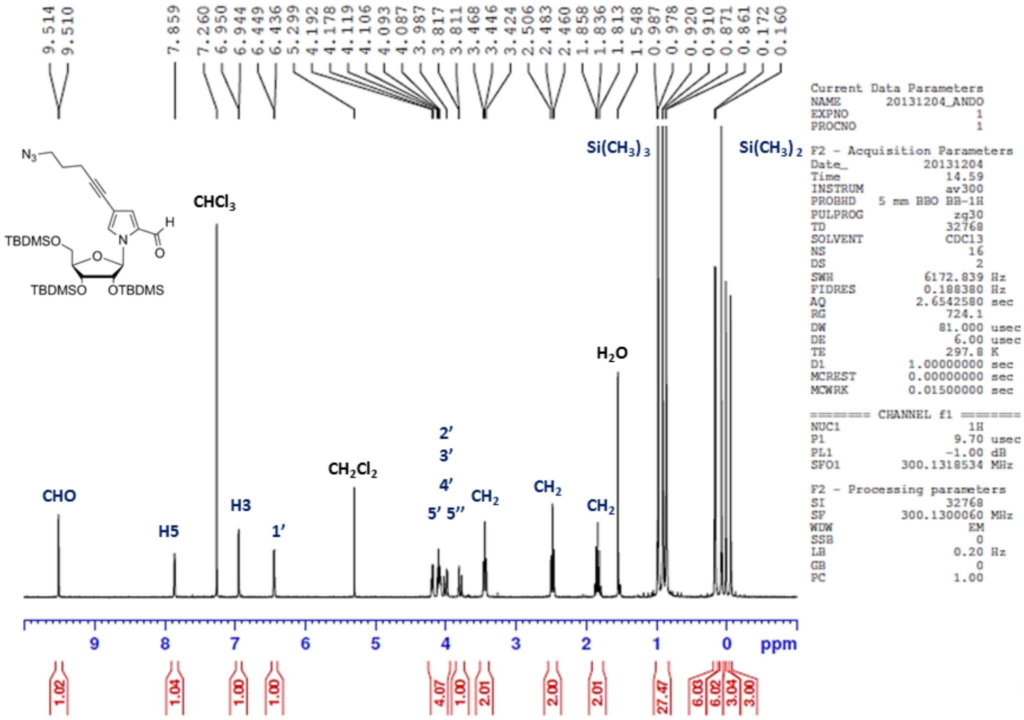

Figure S3. ^{1}H NMR spectrum of compound **4**.

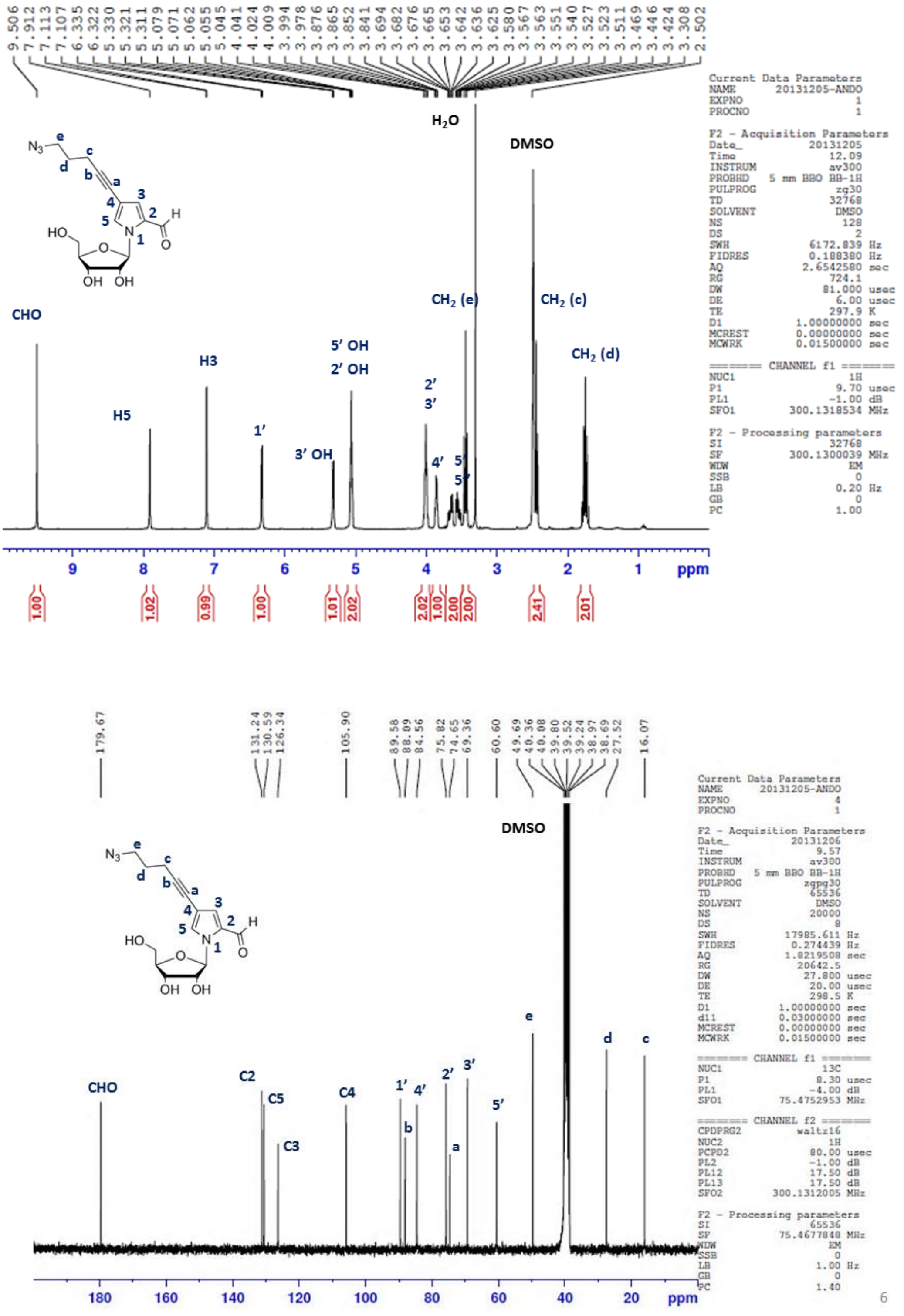

Figure S4. ^{1}H and ^{13}C NMR spectra of compound **5**.

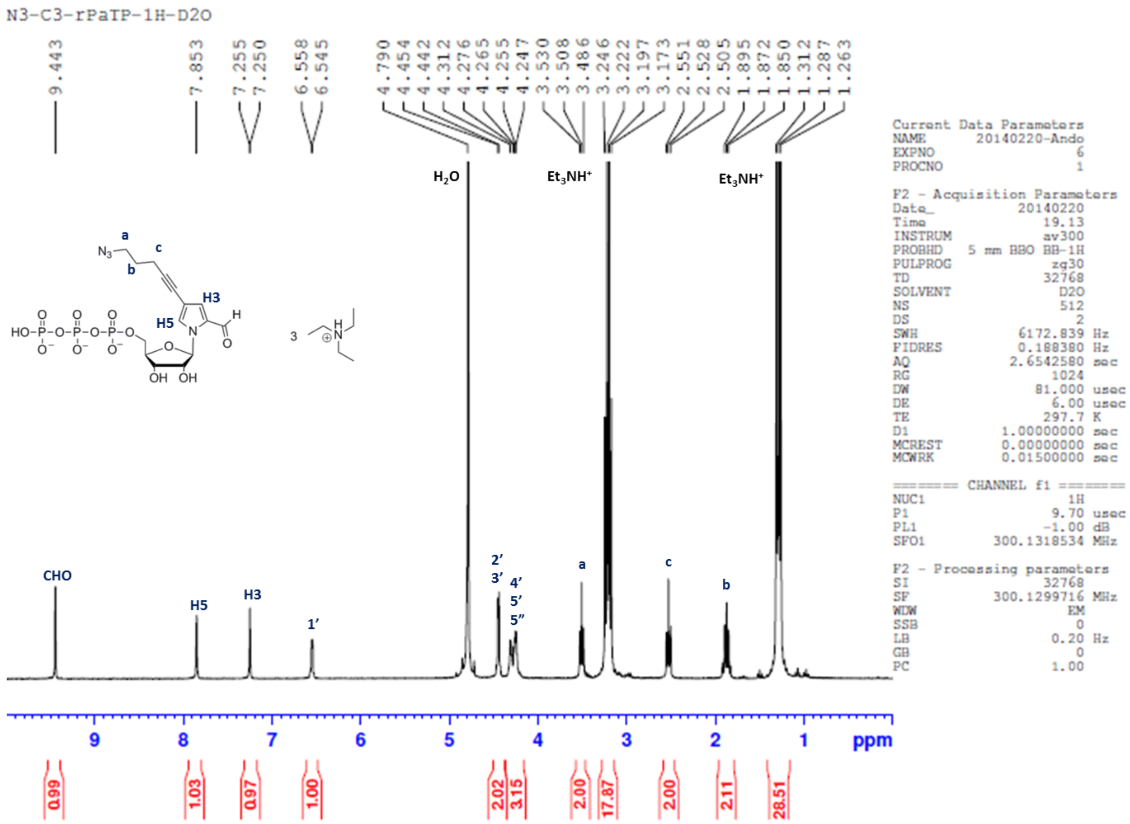

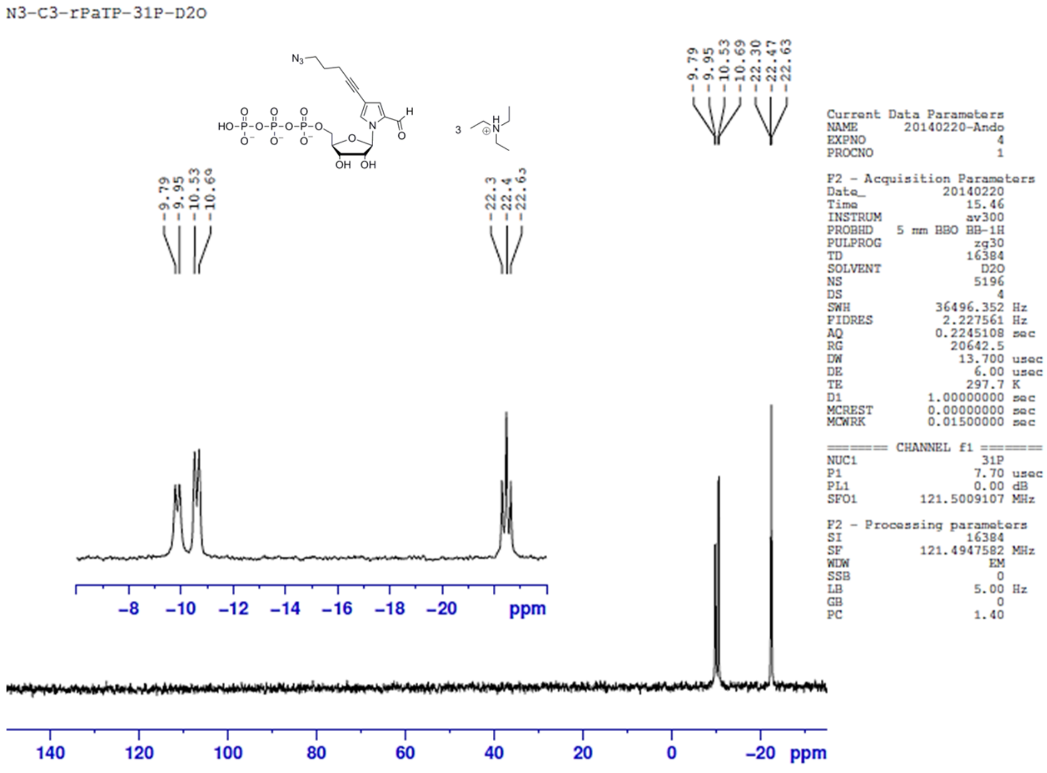

Figure S5. ^{1}H and ^{31}P NMR spectra of compound **6**.

(a)

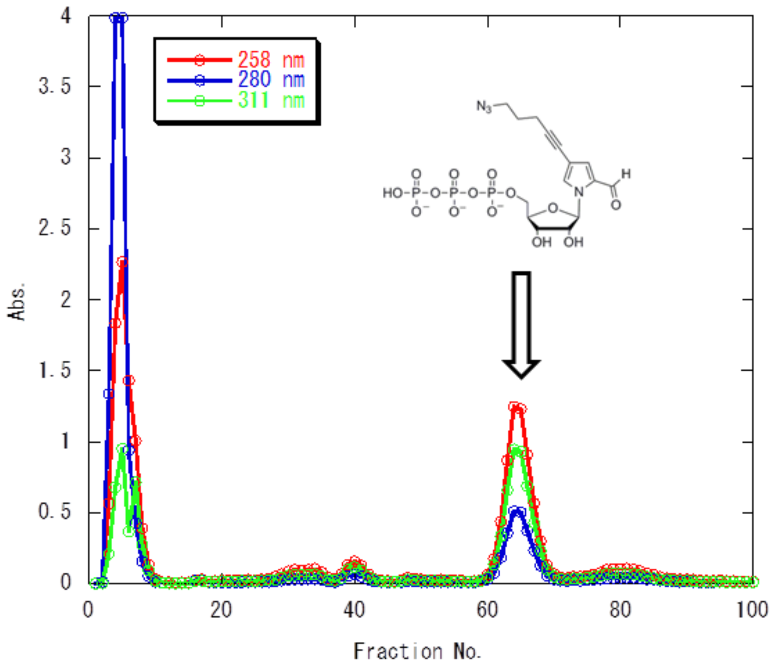

(b)

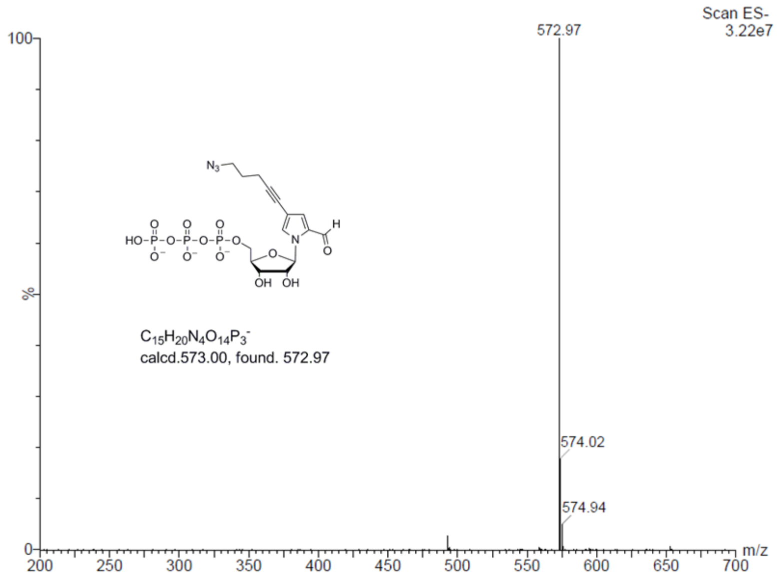

Figure S6. DEAE Sephadex ion-exchange column elution pattern and HRMS spectrum of 5′-triphosphate. (a) DEAE Sephadex ion-exchange column elution pattern of compound **6**. (b) HRMS spectrum of compound **6**.
